# A Meta-Analysis of Timing of Complete Revascularization in Patients with ST-Elevation Myocardial Infarction

**DOI:** 10.3390/jcm13237107

**Published:** 2024-11-24

**Authors:** Michał Kuzemczak, Abdelrahman Mahmoud, Mohammed A. R. Abdellatif, Mohammad Alkhalil

**Affiliations:** 1 Division of Emergency Medicine, Poznan University of Medical Sciences, 61-701 Poznan, Poland; michal.kuzemczak@gmail.com; 2 Department of Cardiology, Biegański Hospital, Medical University of Lodz, 91-347 Lodz, Poland; 3 Department of Interventional Cardiology and Internal Diseases, Military Institute of Medicine—National Research Institute, 05-119 Legionowo, Poland; 4 Faculty of Medicine, Minia University, Minia 2431436, Egypt; abdelrahman.mahmoud20211997@gmail.com; 5 Cardiothoracic Centre, Freeman Hospital, Newcastle-upon-Tyne NE7 7DN, UK; mabdellatif312@gmail.com; 6 Translational and Clinical Research Institute, Newcastle University, Newcastle-upon-Tyne NE1 7RU, UK

**Keywords:** STEMI, complete revascularization, timing, procedural myocardial infarction

## Abstract

**Background***:* Recent randomized clinical trials (RCTs) of STEMI patients with multi-vessel disease (MVD) reported potential superiority of immediate (ICR) vs. staged complete revascularization (SCR). Inherently, the risk of procedural MI is less likely to be detected in ICR patients, and this may have influenced the results. Recently published meta-analyses encompassed observational studies without including STEMI data from the BioVasc trial. The aim of this meta-analysis was to perform an updated comparison of the two strategies in STEMI patients with MVD. **Methods***:* Electronic databases were searched from their inception till August 2024 to identify RCTs assessing CR timing in STEMI patients with MVD. Only studies with an endpoint involving major adverse cardiovascular events (MACE) were included. **Results***:* Six RCTs totaling 2023 patients were included in the analysis. The median time to staged PCI was 19 days. The incidence of MACE (as defined by each study’s protocol) was comparable between the two strategies [RR 0.86, 95% CI (0.58 to 1.27)]. There was also no difference in the risk of non-procedural MI [RR 0.91, 95% CI (0.49–1.67)], death [RR 1.47, 95% CI (0.89–2.44)] and cardiovascular death [RR 1.53, 95% CI (0.79–2.98)]. There was a significant 40% reduction in unplanned revascularization in patients undergoing ICR versus SCR [RR 0.60 (0.40 to 0.89), *p* = 0.01]. **Conclusions***:* ICR reduced the risk of unplanned revascularization compared to SCR but had a comparable effect on MACE, death, cardiovascular death and non-procedural MI. Both strategies are safe in managing patients with acute MI and MVD.

## 1. Introduction

Multivessel coronary artery disease (MVD) is frequently encountered in patients presenting with ST-elevation myocardial infarction (STEMI) who undergo percutaneous coronary intervention (PCI) of the culprit artery responsible for their presentation [1,2]. Previous studies suggested that approximately 50% of STEMI patients have significant lesions in non-infarct-related coronary arteries (non-IRA) and these lesions are directly linked to increased risk of future adverse cardiac events [2,3]. Moreover, complete revascularization (CR) in patients with MVD presenting with STEMI was associated with a significant reduction in cardiovascular mortality compared to a strategy of treating the culprit only [2,3]. Additionally, CR resulted in better functional status and a higher proportion of angina-free patients at a median follow up of three years [4]. The reduction in cardiovascular events alongside symptomatic benefits associated with CR were reflected in current clinical guidelines recommending this strategy in patients with STEMI and concomitant MVD [1,5,6].

Importantly, the optimal timing of CR remains debatable. Broadly, two strategies have emerged to achieve CR by either performing routine PCI during index presentation, referred to as immediate CR (ICR), or as a staged second procedure within the same hospital admission or at follow up (staged CR, SCR) [1,5,6]. Several observational studies assessed the clinical outcomes of the two approaches, i.e., ICR versus SCR, including data from the Complete versus Culprit-Only Revascularization Strategies to Treat Multivessel Disease after Early PCI for STEMI (COMPLETE) trial [3,7]. Recently, two large, randomized studies compared ICR versus SCR in patients presenting with acute myocardial infarction with relatively conflicting results [8,9]. The Multivessel Immediate versus Staged Revascularization in Acute Myocardial Infarction (MULTISTARS-AMI) study highlighted a superiority of ICR compared to the SCR strategy [9]. On the other hand, the immediate versus staged complete revascularization in patients presenting with acute coronary syndrome and multivessel coronary disease (BIOVASC) trial reported non-inferiority results in the primary endpoint between the two approaches in a larger but more heterogenous sample size.^8^ Whilst the difference between these two studies might stem from the variations in the included patients, it is important to highlight that procedural MI is less likely to be diagnosed in patients undergoing ICR, creating an ascertainment bias towards this strategy.

Therefore, the aim of the present updated study-level meta-analysis was to compare the clinical outcomes of patients who exclusively presented with STEMI and MVD and underwent ICR versus SCR and to assess any difference on individual endpoints, including non-procedural MI.

## 2. Methods

The MEDLINE, PubMed and Cochrane Central Register databases were searched from their inception till August 2024 using the following keywords: STEMI, acute MI, multi-vessel, complete revascularization, immediate, staged and timing. Eligible studies were randomized trials involving patients presenting with STEMI who had multi-vessel disease (MVD) as defined by the study’s protocol and were planned for CR. Randomized trials comparing CR to culprit vessel-only revascularization were excluded since the present meta-analysis is focused on the timing of CR instead of contrasting the above-mentioned strategy with a culprit vessel-only approach.

The included studies enrolled patients who were randomized to either immediate (during the index procedure) or staged PCI. The staged PCI group comprised patients who had the procedure during their in-hospital stay or those scheduled for elective PCI following a hospital discharge. Only studies reporting major adverse cardiovascular events (MACE) as the primary endpoint were taken into account, and the clinical endpoint was defined according to the reported events within the included studies.

Studies that exclusively assessing NSTEMI patients were excluded from the present meta-analysis, although studies evaluating both STEMI and NSTEMI cohorts were included. However, data were only included from the STEMI cohort in the current meta-analysis [8,10]. Studies focusing on other primary endpoints such as left ventricular systolic function were also excluded [11]. Importantly, the included studies did not uniformly report all individual endpoints, including spontaneous and procedural MI, but focused on major adverse events (MACE) and death [12].

All the included articles were assessed by two authors (AM, MA) using the prespecified inclusion criteria described above. Neither of them was an investigator in any of the selected studies and any disagreement between the authors was resolved by consensus. Initial search results were screened at a title/abstract level and relevant studies were retrieved for a full review. Full reports were evaluated to confirm inclusion or exclusion in the present meta-analysis. Previously published systematic reviews and meta-analyses on CR were reviewed in order to cross-check the results. The Preferred Reporting Items for Systematic Reviews and Meta-Analyses (PRISMA) guidelines were followed for identifying relevant records and ensuring the proper evaluation of the included studies [13]. In the original trials, patients in the staged PCI group were considered as the control group, while those in the immediate PCI group were defined as the experimental group. The included studies did not specifically report the clinical outcomes of patients undergoing staged PCI during in-hospital stay or a staged elective procedure. Additionally, the timing of staged PCI across the included studies was not uniform and this did not allow sub-group analyses to assess whether there is any meaningful difference in patients undergoing in-hospital versus elective staged PCI.

An Institutional Review Board Statement was not sought nor required given the nature of the current analysis (study-level meta-analysis).

### Statistical Analysis

Continuous data are presented as mean ± SD or as median (range), while categorical variables are presented as percentages, similarly to the data reported in the original studies. Using trial-level data, the treatment effect was reported as a rate ratio (RR) with 95% confidence intervals (CI) (adjusted by person-years to account for potential differences in the included studies’ follow-up). A random-effects model was used to calculate the pooled RRs. This was used to relatively weigh the studies equally since all included studies were randomized trials (the results were consistent when applying fixed effect). Publication bias was evaluated using a funnel plot. The statistical analysis was performed using RevMan software version 5.4 (Cochrane Informatics & Technology, London, UK), and *p* < 0.05 was considered statistically significant.

## 3. Results

**Figure 1:**
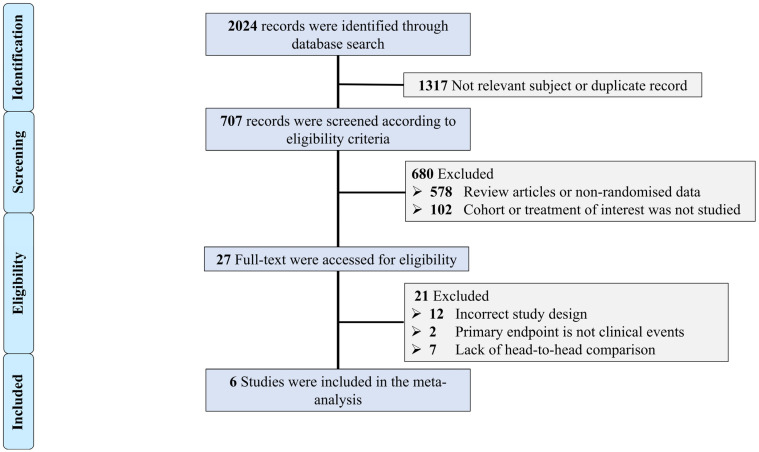
Flow chart of the included studies. Selection process of identified randomized trials evaluating the role of immediate versus staged complete revascularization in patients presenting with ST-segment elevation myocardial infarction and multi-vessel disease.

Using the previously described search strategy, 2024 records were initially identified. The flow chart of screening and including studies are presented in Figure 1. Six randomized trials assessing the role of ICR versus SCR in managing patients presenting with STEMI and MVD were included [9,10,12,14,15,16]. The total number of included patients was 2023, of which 1008 (50%) patients were randomized to ICR compared to 1023 (50%) to SCR. Baseline clinical characteristics are presented in Table 1. All the included studies recruited patients who are relatively young (average age less than 65 years) and patients were predominately male.

**Figure 2:**
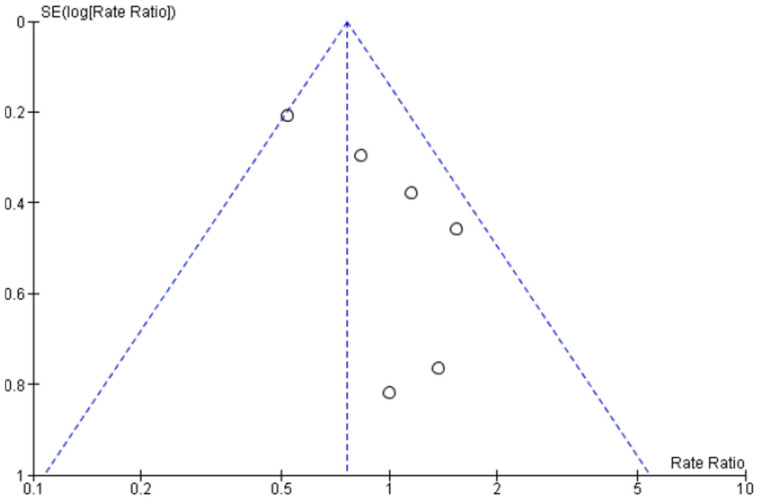
Funnel plot of the included studies. The horizontal axis represents the rate ratio (RR), while the vertical axis reflects the standard error of log RR. The vertical and sloping dotted lines represent the pooled RR and expected 95% CIs for a given standard error (SE), respectively.

The median time to staged PCI was 19 days. In total, there were 65 deaths, of which 38 were from cardiovascular causes. There were 62 MIs, of which 44 were spontaneous MIs and 108 unplanned revascularization events. Risk of publication bias was evaluated by visual assessment of the funnel plot shown in Figure 2.

**Figure 3:**
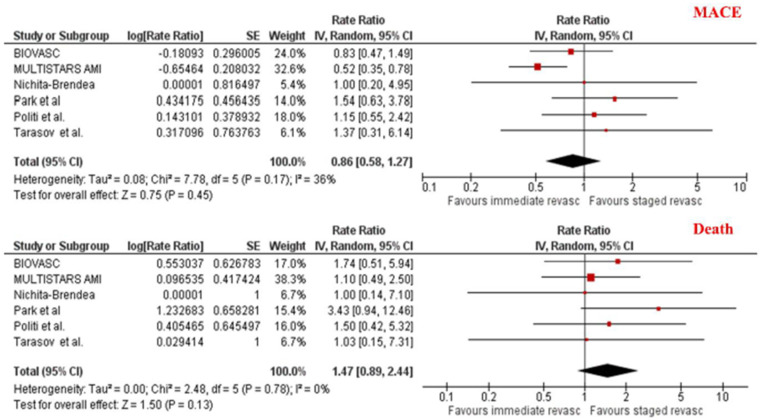
Meta-analysis of MACE and death according to the timing of complete revascularization. Individual and pooled rate ratio of MACE and death with 95% confidence intervals of patients undergoing immediate versus staged complete revascularization [9,10,12,14,15,16].

**Figure 4:**
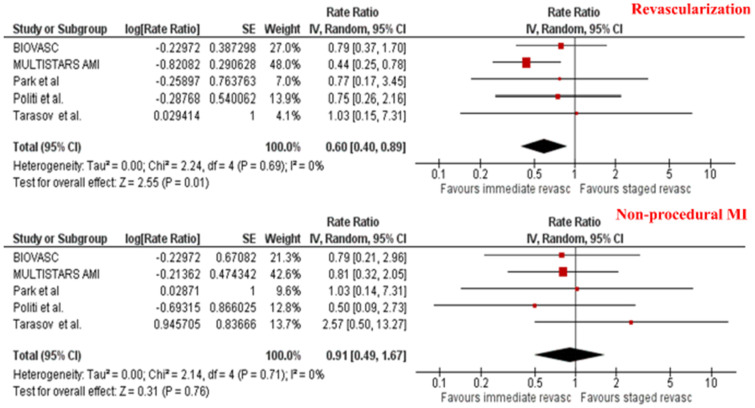
Meta-analysis of unplanned revascularization and non-procedural MI according to the timing of complete revascularization. Individual and pooled rate ratio of unplanned revascularization and non-procedural myocardial infarction with 95% confidence intervals of patients undergoing immediate versus staged complete revascularization [9,10,14,15,16].

The overall incidence of MACE as defined by each study’s protocol was comparable between the two revascularization strategies [8.9% vs. 11.8%; RR 0.86, 95% CI (0.58–1.27), *p* = 0.45]. There was no difference in the risk of death [3.9% vs. 2.6%, RR 1.47, 95% CI (0.89–2.44), *p* = 0.13] and cardiovascular death [2.4% vs. 1.6%, RR 1.53, 95% CI (0.79–2.98), *p* = 0.21] between patients assigned to immediate versus staged CR (see Table 2 and Figure 3). Importantly, there was a significant 40% reduction in the incidence of unplanned revascularization in patients undergoing immediate versus staged CR [4.2% vs. 7.0%, RR 0.60 (0.40–0.89), *p* = 0.01] (see Table 2 and Figure 4). However, the risk of MI was numerically, but not statistically, higher in patients undergoing SCR compared to ICR [2.3% vs. 4.1%, RR 0.58, 95% CI (0.31–1.09), *p* = 0.09]. This numerical difference was attenuated when only assessing spontaneous MI [2.2% vs. 2.4%, RR 0.91, 95% CI (0.49 to 1.67)], highlighting the probability of underdiagnosing procedural MI in patients undergoing ICR (see Table 2 and Figure 4).

The timing of SCR was heterogenous among the studies, with only one study specifying in-hospital CR (Nichita-Brendea et al. [12]). Data on early SCR were reported in two studies, namely the COmplete lesion versus CUlprit lesion revascularization in Acute STEMI patients with MVD undergoing primary PCI with the everolimus-eluting stent (EES, COCUA trial) [14] at 4.4 days and Tarasov et al. [16] at 8.5 days. When data were re-analyzed, excluding studies with early SCR, there was no significant difference between ICR versus SCR in the rate of MACE [RR 0.74, 95% CI (0.46–1.17), *p* = 0.19], death [RR 1.32, 95% CI (0.72–2.40), *p* = 0.37], or non-procedural myocardial infarction [RR 0.74, 95% CI (0.37–1.48), *p* = 0.40], respectively. However, the risk of unplanned revascularization [RR 0.57, 95% CI (0.38–0.87), *p* = 0.009] was significantly reduced in patients who underwent ICR versus late SCR. The number for event rates in patients who underwent early SCR was relatively small (14 events) and precludes any meaningful analysis.

## 4. Discussion

The present study-level meta-analysis provides an updated comparison of randomized trials assessing the role of immediate complete revascularization (ICR) and staged complete revascularization (SCR) strategies in patients presenting with STEMI and multivessel disease. The main findings of this meta-analysis can be summarized as follows: (1) there is no difference in the incidence of MACE, death, or cardiovascular death among patients undergoing ICR and SCR; (2) the risk of MI remains comparable between the groups, particularly after excluding procedural MI and exclusively assessing the incidence of spontaneous MI; and (3) there was a significant reduction in ischemia-driven unplanned revascularization in patients undergoing ICR versus SCR.

Several studies have reported the clinical benefits of achieving CR compared to culprit-lesion-only revascularization [3,17,18,19,20]. This strategy was borne out of the Functional Assessment in Elderly MI Patients with Multivessel Disease (FIRE) trial, and elderly patients assigned to CR sustained 35% risk reduction in the composite of cardiovascular death and myocardial infarction compared to culprit-lesion-only revascularization [21]. Mechanistically, CR might have improved prognosis by prophylactically sealing unstable vulnerable plaque in the non-IRA in patients presenting with STEMI and MVD [22]. This is supported by the optical coherent tomography (OCT) sub-study from the COMPLETE trial highlighting the frequency of complex vulnerable plaque in the non-IRA in this group [22]. However, the role of inflammation in destabilizing coronary plaques in patients with MVD in response to acute MI presentation is another plausible mechanism [23,24].

The timing of performing CR remains controversial. ICR is intuitively associated with a shorter length of in-hospital stay and longer procedural times, and is associated with increased contrast volume. The latter disadvantages might be offset if ICR may result in a reduction in future adverse events. However, previous meta-analysis highlighted comparable outcomes irrespective of the setting of CR, i.e., during index presentation, in-hospital staged procedure, or elective outpatient setting [25]. Recently, the MULTISTAR and BIOVASC trials were designed to address the issue of timing of CR [8,9]. The BIOVASC trial recruited 1362 patients with acute MI and MVD, but only recently reported the clinical outcomes of the STEMI group [10]. Interestingly, and in contrast to the MULRI-STAR study, Scarparo et al. reported similar outcomes in patients receiving ICR versus SCR.

Given the inconsistency in establishing any difference between the two strategies of achieving CR, three meta-analyses were conducted assessing the role of ICR versus SCR [25,26,27]. However, all of these meta-analyses included observational studies with inherent methodological limitations yielding lower quality evidence and challenging any definitive conclusions in establishing the optimal timing of CR [25,26,27]. The study by Maamoun et al. [28] is a quasi-randomized study whereby the authors recruited patients presenting with STEMI and MVD in the first year of the study and underwent SCR, compared with those presenting in the second year who underwent ICR [28]. Similarly Wood et al. assessed the outcomes of in-hospital CR versus elective staged CR in the COMPLETE trial, but the decision to assign patients to the CR strategy was based on the operator’s discretion and not subjective to randomization [7]. Moreover, the BIOVASC trial only recently reported the clinical outcomes of its STEMI group [10]. The heterogeneity of including both STEMI and NSTEMI patients may have an impact on the final results. This is important, given the discordant results within the BIOVASC study according to the type of acute MI presentation. Elscot et al. [29] reported a significant reduction in MIs, even after excluding procedural MI, and unplanned revascularization in patients presenting with NSTEMI and who underwent ICR, compared to SCR, in the BIOVASC trial. There was no difference in all-cause death, cardiovascular death or MACE between the two strategies [29]. In contrast, Scarparo et al. [10] highlighted comparable outcomes of ICR versus SCR in the incidence of death, MI, unplanned revascularization and MACE in the STEMI group. Our meta-analysis applied more stringent criteria and only included truly randomized studies that exclusively evaluated patients presenting with STEMI with MVD and compared the clinical outcomes of ICR versus SCR.

In the current study, we elected to assess the difference in spontaneous myocardial infarctions according to the CR strategies. Procedural MI in patients presenting with STEMI who underwent ICR is likely to be underdiagnosed and its incidence to be underestimated. Ascertaining the incidence of procedural MI in this group is inherently flawed, and it remains to be determined and is probably indistinguishable from the index procedure for acute MI presentation [30]. Subsequently, our meta-analysis did not demonstrate any difference in the incidence of total MI or spontaneous MI. This in contrast to other meta-analyses reporting a significant reduction in MI in patients who underwent the ICR strategy [25,26]. Such a phenomenon is related to the difference in the definition of MI and the heterogeneity of included studies in each meta-analysis. Importantly, the differential prognostic impact of procedural versus spontaneous MI on mortality is well-established [31,32]. Whilst there is a relevant increase in mortality after spontaneous MI, this phenomenon is not evident after procedural MI with either percutaneous coronary intervention of coronary artery bypass grafts (CABG) [31,32]. More recently, the prognostic relevance of the used cut-off of biomarkers elevation to define procedural MI was highlighted from a large real-world data of more than 10,700 patients [33]. In this study, Spirito et al. reported that 1 year mortality was significantly increased after procedural MI if troponin elevation was more than 35 times the upper reference level (URL). In contrast, procedural MI with troponin elevation of less than 35 times the URL was not associated with worsening mortality. This becomes important given that procedural MI in the included studies have predominately used the Third Universal Definition of Myocardial Infarction which defines procedural MI as a biomarker elevation of more than 5 times URL only [34].

The incidence of death or cardiovascular death was comparable across all reported meta-analyses. Likewise, the risk of unplanned revascularization was consistently lower in patients assigned to ICR. This observation may be a true effect of the ICR strategy eliminating residual ischemia of obstructive non-IRA lesions. Nonetheless, the lack of blinding in the reported randomized studies cannot exclude information bias, subjecting patients in the SCR strategy to a lower threshold of intervention during any subsequent medical encounter. On the other hand, there may be a plausible biological explanation, beyond information bias, which could explain the increased risk of unplanned revascularization in patients with SCR. Following acute MI, there is an inflammatory response that may destabilize non-culprit coronary plaques, triggering early remote plaque rupture and chest pain presentation [23]. However, this mechanism should be mirrored by an increase in spontaneous MI in patients assigned to SCR which was not evident in the included studies.

The current meta-analysis only included patients presenting with STEMI and excluded those with NSTEMI. There is a differential pathophysiological process, including the inflammatory response between the two main subtypes of acute MI [35,36]. This may be associated with different responses to CR accordingly. In fact, data from the BIOVASC study highlighted similar clinical outcomes in patients presenting with STEMI and undergoing ICR versus SCR [10]. On the other hand, data from the same study evaluating the timing of CR in patients presenting with NSTEMI highlighted a significant reduction in MI and unplanned revascularization in patients undergoing ICR versus SCR [29]. Moreover, the role of functional or angiographic assessment in identifying and treating significant non-culprit coronary lesions may be different in patients with STEMI versus NSTEMI [37]. Unlike patients presenting with NSTEMI, it appears that functional assessment did not help reducing event rates compared to angiographic guidance in patients with STEMI and multivessel disease undergoing CR [38,39]. 

## 5. Limitations

The current meta-analysis included study-level and not individual-level data; therefore, assessment of clinical outcomes according to certain anatomical or procedural characteristics was not possible. Although there was some heterogeneity in the primary endpoint of the included studies, they were all randomized trials and their primary endpoint was major adverse cardiovascular events. Finally, the majority of the included studies reported clinical outcomes up to 12 months and, therefore, the impact of ICR on long-term follow up cannot be ascertained.

## 6. Conclusions

ICR reduced the risk of unplanned revascularization compared to SCR but had comparable effect on MACE, death, cardiovascular death and non-procedural MI. Both strategies can be considered safe in managing patients with acute MI and MVD. It remains plausible that a tailored approach of timing complete revascularization may provide an optimal approach for certain individuals.

## Figures and Tables

**Table 1:** Baseline characteristics of the included studies.

Study	Number of Patients	Age	Male (%)	Timing to Staged PCI (days)	Primary Endpoint	Duration of Follow Up	Male %	DM %	Hypertension	Current Smoker	PAD %	Previous MI	Previous PCI	Family History of CVD	LVEF %	Anterior STEMI/LAD IRA %	Antiplatelets %ASA/Clopidogrel/Ticagrelor/Prasugrel	3-Vessel Disease %
Politi et al. (2010) [15]	130	65	78	57	Cardiac or non-cardiac death, in-hospital death, re-infarction, re-hospitalization for ACS and repeat coronary revascularisation.	30 months	77.6	19.1	57.9	-	-	-	-	-	45.2	44.2	94/92.1/-/-	32.2
Tarasov et al. (2017) [16]	136	59	64	8.6	Cardiac death, myocardial infarction, target vessel revisualization	6 months	64	23.6	91	-	25.8	7.9	-	-	51.6	38.2	-/-/-	45
Nichita-Brendea et al. (2021) [12]	100	-	73	Within 48–72 h of index presentation	Death, stroke, myocardial infarction and symptom induced revascularization	12 months	73	23	44	46	-	-	-	-	43.6	40	-/-/-	23 **
Park et al. (2023) [14]	209	63	81	4.4	Death, recurrent myocardial infarction and revascularization	12 months	81.3	37.8	49.8	39	-	1	1	2	51.4	46.5	100/90/-	18.7
Stähli et al. (2023) [9]	840	65	79	37	Death from any cause, nonfatal myocardial infarction, stroke, unplanned ischemia-driven revascularization, or hospitalization for heart failure	12 months	78.8	15.6	52.4	34.4	2.3	5.7	6.7	26.4	-	40.4	- *	15.6
Scarparo et al. (2024) [10]	608	63	79	19	All-cause mortality, myocardial infarction, any unplanned ischaemia-driven revascularisation, or cerebrovascular events	12 months	79.3	16	43.6	36	3	6.6	10	30.6	-	37.4	-/10.2/67/23	20

* data available only on patients who experienced a primary endpoint, not for all the included patients. ** the percentage of patients with 3-vessel disease including IRA; patients with 4- and 5-vessel disease were calculated separately in the study and comprised 16% and 3% of the included subjects, respectively; ACS: acute coronary syndrome.

**Table 2:** Clinical outcomes of patients with acute myocardial infarction and multi-vessel disease, stratified according to the timing of CR strategy.

	Event Rate (Immediate Revascularization) (n = 1008)	Event Rate (Staged Revascularization) (n = 1015)	Rate Ratio (95% Confidence Interval)	*p* Value
Death	39 (3.9%)	26 (2.6%)	1.47 (0.89–2.44)	0.13
Cardiovascular death ^⁋^	23 (2.4%)	15 (1.6%)	1.53 (0.79–2.98)	0.21
Myocardial infarction ^⁋^	22 (2.3%)	40 (4.1%)	0.58 (0.31–1.09)	0.09
Unplanned Ischaemia-driven revascularization ^⁋^	40 (4.2%)	68 (7.0%)	0.60 (0.40–0.89)	0.01
Myocardial infarction (excluding procedural) ^⁋^	21 (2.2%)	23 (2.4%)	0.91 (0.49–1.67)	0.76
Major adverse cardiovascular events *	90 (8.9%)	120 (11.8%)	0.86 (0.58–1.27)	0.45

^⁋^ Events rate was calculated from 5 studies [9,10,14,15,16]. Brendea et al. [12] only reported on the incidence of death and major adverse cardiovascular events. * defined as the primary endpoint in each study’s protocol.

## Data Availability

The original contributions presented in the study are included in the article, further inquiries can be directed to the corresponding authors.
